# The effect of moral distress on emergency nurses’ burnout: mediating role of moral resilience and moderating role of moral courage

**DOI:** 10.3389/fpubh.2026.1829866

**Published:** 2026-05-18

**Authors:** Qizhe Dong, Jintao Bao, Yuyan Yang, Shilin Ma, Fengming Huang

**Affiliations:** 1 School of Nursing, Ningxia Medical University, Yinchuan, China; 2 Department of Emergency, General Hospital of Ningxia Medical University, Yinchuan, China

**Keywords:** emergency, job burnout, moral distress, moral resilience, nurse

## Abstract

**Background:**

The burnout rate among emergency nurses in China has reached 87.6%. This alarmingly high prevalence of burnout is driving a substantial number of experienced emergency nurses to leave their positions, thereby posing a serious threat to the sustainable and healthy development of the healthcare system. Moral distress represents a major modifiable risk factor for burnout. The present study aims to explore the correlation between moral distress and burnout, whether moral resilience could act as a mediator of the relationship, and whether moral courage moderates the mediating effect among emergency nurses.

**Methods:**

This study employed a cross-sectional design and was carried out among emergency department nurses from seven tertiary Class A hospitals in the Ningxia Hui Autonomous Region, China, in August 2025. Survey instruments included a demographic questionnaire, the Maslach Burnout Inventory-Human Services Survey (MBI-HSS), the Chinese version of the Rushton Moral Resilience Scale, the Moral Distress Scale-Revised (MDS-R), and the Nurses’ Moral Courage Scale (NMCS). Descriptive analysis and Pearson correlation analysis were performed using SPSS 27.0. The PROCESS macro was applied, with Model 4 for mediation analysis and Model 7 for moderated mediation analysis.

**Results:**

The total scores were as follows: moral distress (45.63 ± 34.18), moral resilience (47.75 ± 6.51), moral courage (71.38 ± 15.24), and burnout (56.09 ± 17.06). Moral distress was positively correlated with burnout among emergency nurses. Moral resilience mediated the association between moral distress and burnout, accounting for 24.8% of the total effect. Moral courage moderated the path from moral distress to moral resilience.

**Conclusion:**

This study found that among emergency nurses, moral distress was positively correlated with burnout; moral resilience mediated the relationship between moral distress and burnout; and moral courage moderated the relationship between moral distress and moral resilience.

## 1 Introduction

Nurses represent a high-risk group for burnout, with an incidence rate as high as 64.5% (1). Emergency nurses care for critically ill patients daily and are required to provide precise and rapid management of their conditions. Factors such as frequent night shifts, heavy workloads, and occupational exposure increase nurses’ susceptibility to burnout (2, 3). Research has indicated (4) that the incidence of burnout among emergency nurses in China reaches 87.6%.

Burnout leads to reduced work efficiency, poor nursing quality, and increased adverse event rates among nursing staff, negatively impacting their physical and mental health (5, 6). Previous research findings indicate that moral distress experienced by nurses is a significant factor influencing burnout among emergency nurses (7). Moral distress refers to the negative psychological experience where an individual is aware of the corresponding behavioral norms but, for various reasons, is unable to act in accordance with them (8). Nurses choose to proactively avoid situations to prevent direct conflicts arising from moral distress. When nurses reach the point where the psychological distress caused by moral distress becomes overwhelming, they are more likely to develop burnout (9).

Nursing moral resilience is defined as the ability of nurses to maintain or restore their own moral integrity when confronted with complex moral situations, moral distress, or stressors (10). Li et al. (11) found that nurses’ moral resilience is negatively correlated with burnout, and moral resilience is crucial for mitigating burnout. Meanwhile, research by Asadi et al. (12) confirmed that moral distress is negatively correlated with moral resilience. Currently, there is limited research on the relationship among moral distress, moral resilience, and burnout. Accordingly, Hypothesis 1 is proposed: Moral resilience mediates the relationship between moral distress and burnout among emergency nurses. Moreover, moral courage and moral resilience jointly serve to uphold professional integrity in the face of moral distress (13), and nurses with higher moral courage are more likely to possess robust moral resilience (5, 6), thereby alleviating burnout. Based on these findings, Hypothesis 2 is proposed: Moral courage moderates the relationship between moral distress and moral resilience among emergency nurses.

**Figure 1:**
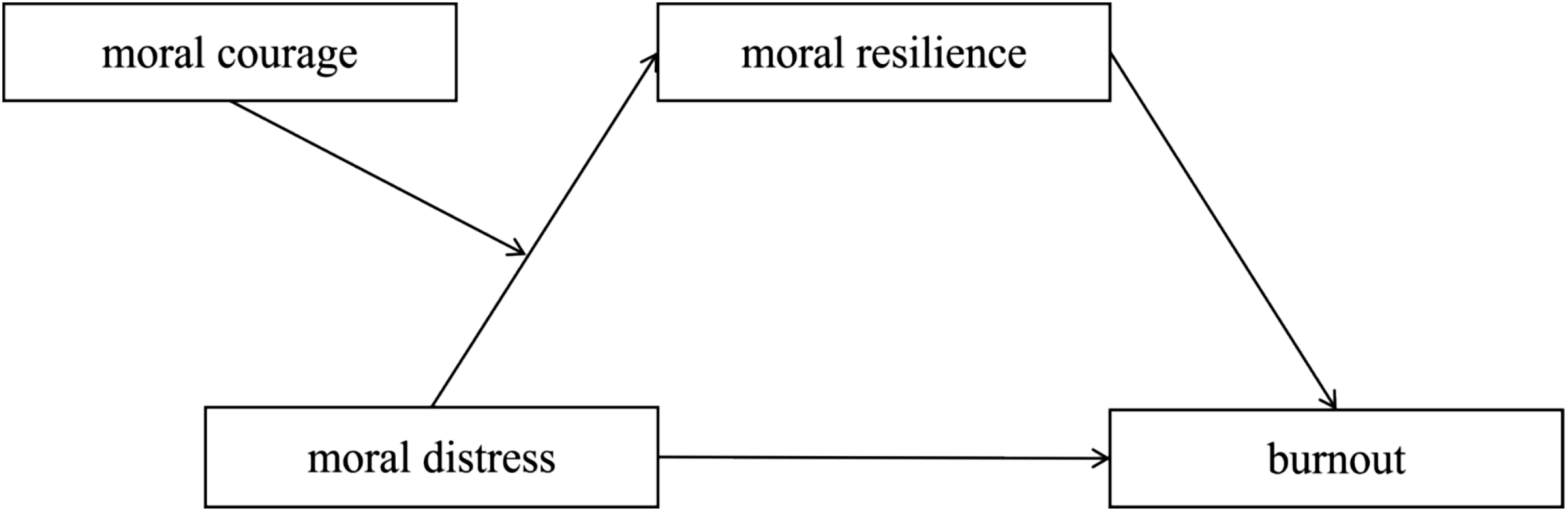
The hypothesized moderated mediation model.

According to the Comer and Sekerka (14) model of enduring moral courage, moral courage is the authentic expression of authenticity in the face of discomfort arising from dissent, opposition, or exclusion. Through psychological and behavioral factors, moral courage can be transformed into enduring moral courage—a specific form of moral resilience—thereby helping individuals alleviate burnout. An intermediary mediation model was proposed to examine the relationship between moral distress and burnout among emergency nurses, as shown in Figure 1. Exploring the relationship between moral distress and burnout among emergency nurses serves two purposes: it enriches and advances the theoretical understanding of the relationship between moral distress and burnout, while also providing valuable insights for nursing administrators seeking to alleviate burnout levels among emergency nurses.

## 2 Method

### 2.1 Aims

The purpose of this study is to examine the relationship between moral distress and burnout among emergency nurses, as well as the mediating role of moral resilience between moral distress and burnout, and the moderating role of moral courage between moral distress and moral resilience.

### 2.2 Design

This study employed a cross-sectional, online questionnaire survey design. The results were reported according to the Strengthening the Reporting of Observational Studies in Epidemiology (STROBE) guidelines (15).

### 2.3 Participants

From August to September 2025, nurses from the emergency departments of seven tertiary Class A hospitals in Ningxia Hui Autonomous Region, China, were selected as study subjects using convenience sampling. Inclusion criteria: ① Years of experience in the emergency department ≥ 1 year; ② Currently employed and not in the process of resigning; ③ Voluntarily agreed to participate in the study and signed the informed consent form.

The exclusion criteria were: (1) Nurses who withdrew from the study due to reasons such as further education, maternity leave, or personal leave.

### 2.4 Samples

This study was designed as a cross-sectional investigation. The sample size was calculated using the cross-sectional formula *n* = ( $\mu_{\alpha /2}$ *σ*/*δ*)^2^ With *α* = 0.05, $\mu_{\alpha /2}$ = 1.96, and *σ* = 8.76 based on previous studies, and setting *δ* = 1, we obtained *n* = (1.96 × 8.76/1)^2^ ≈ 295. Accounting for a 10% non-response rate, the study required a minimum sample size of 325 cases. Ultimately, 333 cases were enrolled, meeting the sample size requirement.

### 2.5 Measures

#### 2.5.1 Demographic questionnaire

The questionnaire was designed by the researcher based on a systematic review of relevant literature and the results of group discussions within the research team. It encompasses 14 variables covering the research subjects’ gender, age, working years, marital status, educational level, professional title, labor and personnel relations, average number of night shifts, participation in hospital ethics training, average monthly income, only child status, fertility status, department, and health status.

#### 2.5.2 MBI-human service survey, (MBI-HSS)

The Maslach Burnout Inventory, developed jointly by Maslach and Jackson and validated by Feng et al. (16) among Chinese nurses, demonstrates excellent reliability and validity. This scale comprises three dimensions and 22 items: Emotional Exhaustion (9 items), Depersonalization (5 items), and Personal Achievement (8 items). The Personal Achievement dimension (items 4, 7, 9, 12, 17, 18, 19, 21) is reverse-scored. After reverse-scoring the personal achievement dimension, a 7-point Likert scale is applied, ranging from “never” to “every day,” with scores ranging from 0 to 6 points, respectively. Higher scores indicate more severe burnout. The total score range is 0–132 points, with a Cronbach’s *α* coefficient of 0.905.

#### 2.5.3 Moral distress scale (MDS-R)

The scale was developed by Corley et al. (17), and later revised by Hamric and Blackhall (18), and its Chinese version was validated by Sun (19). The scale comprises four dimensions: Individual Responsibility (8 items), Not in the Patient’s Best Interest (5 items), Value Conflicts (6 items), and Harming in the Patient’s Interest (3 items), totaling 22 items. Each item is evaluated based on two dimensions: frequency of occurrence and level of distress, using a 5-point scoring system. Frequency is rated from “never” to “very frequently,” scoring 0 to 4 points, respectively. Distress is rated from “none” to “severe,” scoring 0 to 4 points, respectively. The final score for each item is the product of its frequency and distress score. The total scale score ranges from 0 to 352, with higher scores indicating greater levels of moral distress among survey participants. In this study, the Cronbach’s *α* coefficient for the scale was 0.879.

#### 2.5.4 Rushton moral resilience scale Chinese version

The scale was developed by Heinze et al. (20), and translated and revised by Tian et al. (21). This scale comprises three dimensions: the Ability to Cope with Moral Adversity Flexibly (5 items), Relational Moral Soundness (6 items), and Moral Efficacy (6 items), totaling 17 items. Each item uses a 4-point Likert scale from 1 to 4, ranging from “strongly disagree” to “strongly agree.” The total score ranges from 17 to 68, with higher scores indicating stronger moral resilience. Items 2, 4, 5, 6, 8, 10, 11, 13, 14, 15, and 16 are reverse-scored. In this study, the Cronbach’s *α* coefficient for this scale was 0.811.

#### 2.5.5 Nurses moral courage scale (NMCS)

The scale was developed by Numminen et al. (22), and translated by Wang et al. (23). It encompasses four dimensions: Moral Integrity (7 items), Commitment to Good Care for Patients (5 items), Compassion and True Presence with Patients (5 items), and Moral Responsibility (4 items), totaling 21 items. Each item is scored on a 5-point scale, ranging from 1 to 5 (1 = “does not describe me at all”, 5 = “describes me excellently”). The total scale score ranges from 21 to 105, with higher scores indicating greater moral courage among nurses. In this study, the Cronbach’s *α* coefficient for the scale was 0.905.

### 2.6 Data collection

The researchers developed an online survey on the WenjuanXing platform. Prior to the survey, they explained the purpose and significance of the study to the head nurses of the emergency departments at each hospital and obtained their consent. The head nurses then shared the survey in their department’s work group, emphasizing the principles of voluntary participation, anonymity, and informed consent. Participants could access the questionnaire via the link or QR code. They had to read and agree to the informed consent form before proceeding to the next step. They could withdraw from the survey at any time. If they had any questions while completing the questionnaire, they could contact the researchers by phone or WeChat. To ensure the quality of collected data, each IP address was allowed to complete the survey only once. Prior to the formal study, 20 emergency nurses who met the inclusion criteria were invited to participate in a pilot study; the survey data from these participants were not included in the final study. Preliminary research indicated that all items could be understood correctly and required no revision. A total of 373 questionnaires were collected for this study, with an average completion time of 10 to 15 min. Invalid questionnaires were identified according to two criteria: (1) excessively fast completion (i.e., <10 min); and (2) illogical response patterns, defined as mechanically repetitive answers (i.e., uniform ratings across all Likert scale items). After excluding 40 invalid questionnaires meeting the above criteria, 333 valid questionnaires were ultimately retained, resulting in a valid response rate of 89.27%.

### 2.7 Ethical considerations

This study was approved by the Ethics Committee of Ningxia Medical University General Hospital (Ethics Approval Number: KYLL-2026-0341). All participants provided informed consent and participated voluntarily. All data were used only for this study and kept strictly confidential.

### 2.8 Data analysis

### 2.9 Statistical analysis

Descriptive analysis of the data was performed using SPSS 27.0. The Harman single-factor test was employed to examine common method bias. Moral distress, moral resilience, moral courage, and burnout were analyzed using Pearson correlation. PROCESS macro Version 4.1 was used to test mediation and moderation effects: Model 4 examined the mediating effect of moral resilience between moral distress and burnout; Model 7 examined the moderating effect of moral courage on the relationship between moral distress and moral resilience. The bootstrap method with 5,000 resamples was used to estimate 95% confidence intervals (CIs) for indirect effects; an indirect effect was considered significant if the CI did not include zero.

## 3 Result

### 3.1 Common method Bias

The Harman single-factor test was employed to examine the presence of common method bias. Results indicated that 17 factors had eigenvalues greater than 1, with the largest factor explaining 16.8% of the variance (<40%). Therefore, no significant common method bias was identified in this study.

### 3.2 Descriptive statistics of demographic data

A total of 373 questionnaires were collected for this survey. After excluding 40 questionnaires that were completed in less than 10 min or contained mechanical responses, 333 valid questionnaires were retained, resulting in a valid response rate of 89.27%.

Among the 333 emergency nurses, 264 (79.3%) were female; 220 (66.0%) were aged 31–40 years; 113 (33.9%) had 11–15 years of emergency nursing experience; 274 (82.2%) were married; 318 (95.4%) held bachelor’s degrees; 176 (52.8%) held the title of supervisor nurse; 300 (90.1%) were contract or personnel agency staff; 222 (66.6%) worked 5–10 night shifts monthly; 262 (78.6%) had participated in hospital ethics courses; 262 (78.6%) had an average monthly income of 5,001–10,000 RMB; 290 (87.1%) were non-only children; 138 (41.4%) had two children; 160 (48.0%) worked in the resuscitation zone; and 168 (50.5%) reported good health. Data are presented in Table 1.

**Table 1:** Sociodemographic information of the participants (*n* = 333).

Variable	*n* (%)
Gender	
Male	69 (20.7%)
Female	264 (79.3%)
Age [years]	
21–30	67 (20.1%)
31–40	220 (66.0%)
41–50	39 (11.7%)
51–60	7 (2.2%)
Years of working	
1–5	66 (19.8%)
6–10	98 (29.4%)
11–15	113 (33.9%)
16–20	35 (10.5%)
21–25	12 (3.6%)
>25	9 (2.8%)
Marital status	
Unmarried	51 (15.3%)
Married	274 (82.2%)
Divorced/widowed	8 (2.5%)
Educational level	
Below bachelor’s degree	13 (3.9%)
Bachelor’s degree	318 (95.4%)
Above bachelor’s degree	2 (0.7%)
Professional title	
Nurse	28 (8.4%)
Senior nurse	118 (35.4%)
Supervisor nurse	176 (52.8%)
Deputy/director nurse	11 (3.4%)
Labor and personnel relations	
Formally in the compilation	33 (9.9%)
Contract system/personnel agency	300 (90.1%)
Average number of night shifts (pieces)	
<5	89 (26.7%)
5–10	222 (66.6%)
>10	22 (6.7%)
Participation in hospital ethics course	
Yes	262 (78.6%)
No	71 (21.4%)
Average monthly income (RMB)	
<5,000	32 (9.6%)
5,001–10,000	262 (78.6%)
10,001–20,000	39 (11.8%)
Single child	
Yes	43 (12.9%)
No	290 (87.1%)
Fertility status	
Be childless	44 (13.2%)
1 child	115 (34.5%)
2 children	138 (41.4%)
>2 children	36 (10.9%)
Department	
Resuscitation zone	160 (48.0%)
Observation zone	28 (8.4%)
Emergency ward	53 (15.9%)
EICU	65 (19.5%)
General outpatient area	27 (8.2%)
State of health	
Generally	165 (49.5%)
Good	168 (50.5%)

### 3.3 Descriptive statistics and correlation analysis of variables

The mean, standard deviation, and correlation coefficient results for the variables in this study are presented in Table 2. Emergency nurses scored 45.63 ± 34.18 on the moral distress scale, 47.75 ± 6.51 on the moral resilience scale, 71.38 ± 15.24 on the moral courage scale, and 56.09 ± 17.06 on the burnout scale. Pearson correlation analysis revealed that moral distress was negatively correlated with moral resilience (*r* = −0.238, *p* < 0.01) and moral courage (*r* = −0.207, *p* < 0.01), while was positively correlated with burnout (*r* = 0.201, *p* < 0.001). Moral resilience was positively correlated with moral courage (*r* = 0.256, *p* < 0.01) and was negatively correlated with burnout (*r* = −0.248, *p* < 0.01). Furthermore, moral courage was negatively correlated with burnout (*r* = −0.411, *p* < 0.01), as shown in Table 2.

**Table 2:** Descriptive statistics and correlation analysis of each variable.

Variables	*M* ± SD	1	2	3	4
1. Moral distress	45.63 ± 34.18	1	–	–	–
2. Moral resilience	47.75 ± 6.51	−0.238^**^	1	–	–
3. Moral courage	71.38 ± 15.24	−0.207^**^	0.256^**^	1	–
4. Burnout	56.09 ± 17.06	0.201^***^	−0.248^**^	−0.411^**^	1

^**^p < 0.01, ^***^p < 0.001.

### 3.4 Mediating effect analysis

To assess the mediating effect of moral resilience between moral distress and burnout among emergency nurses, Model 4 from PROCESS was employed. After controlling for factors such as age and gender, the results revealed a significant positive correlation between moral distress and burnout (*β* = 0.101, *t* = 3.746, *p* < 0.01). Moral distress was negatively correlated with moral resilience (*β* = −0.456, *t* = −4.456, *p* < 0.001). Moral resilience was negatively correlated with burnout (*β* = −5.513, *t* = −3.862, *p* < 0.001). Furthermore, after including the mediating variable, the direct effect of moral distress on burnout remained significant (*β* = 0.076, *t* = 2.792, *p* < 0.01). As shown in Table 3. Moreover, the upper and lower bounds of the bootstrap 95% CI for both the direct effect of moral distress on burnout and the mediating effect of moral resilience did not include zero, indicating that the mediating effect was significant. The mediating effect accounted for 24.8% of the total effect. As shown in Table 4. Therefore, Hypothesis 1 was supported.

**Table 3:** The mediating effect of moral resilience on the relationship between moral distress and burnout.

Variables	Moral resilience			Burnout			Burnout		
	*β*	*t*	*p*	*β*	*t*	*p*	*β*	*t*	*p*
Moral distress	−0.456	−4.456	<0.001	0.101	3.746	<0.01	0.076	2.792	<0.01
Moral resilience							−5.513	−3.862	<0.001
*R* ^2^	0.058			0.044			0.085		
*F*	5.104			3.793			6.147		

**Table 4:** Direct and indirect effects of moral distress on burnout.

Variables	*β*	SE	LICI	ULCI	Ratio of effect value
Total effect	0.101	0.270	0.048	0.154	100
Direct effect	0.076	0.272	0.022	0.129	75.2
Indirect effect	0.025	0.087	0.010	0.044	24.8

### 3.5 Moderated mediation effect analysis

**Figure 2:**
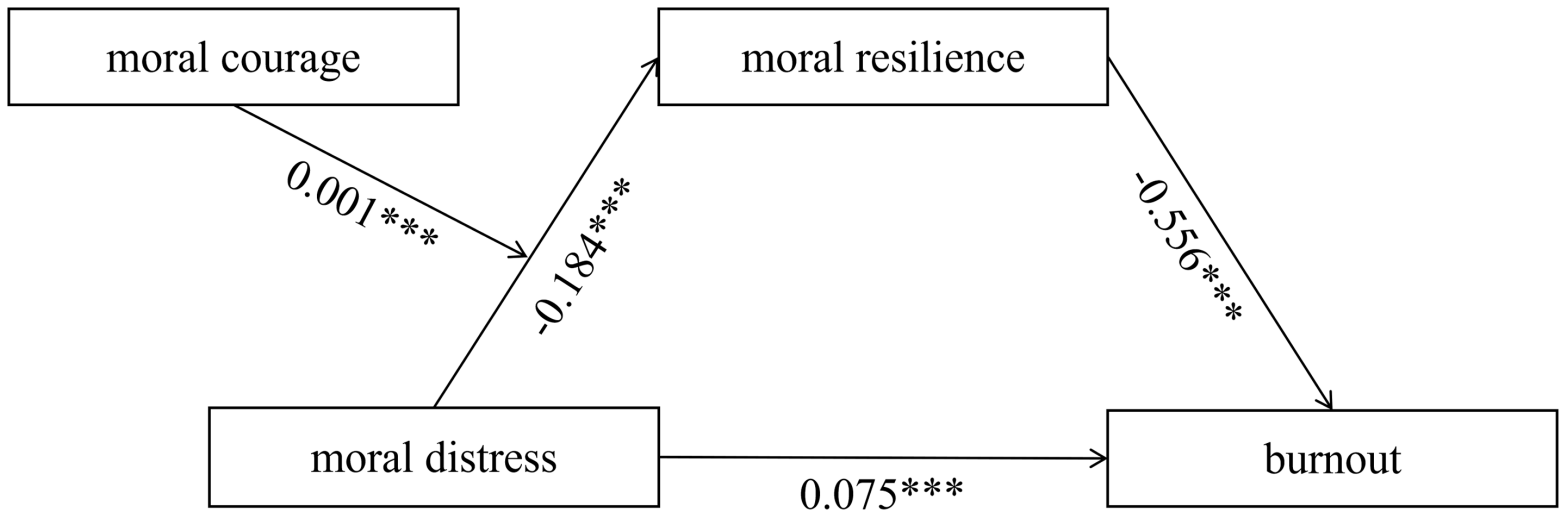
The final moderated mediation model (^***^p < 0.001).

**Figure 3:**
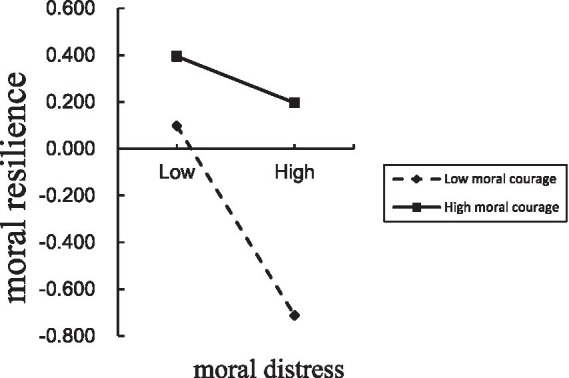
The moderating role of moral courage in mediating model.

PROCESS macro Model 7 was used to test the moderating effect of moral courage. Moral courage was introduced as a moderator into the moderation model, while controlling for factors such as age and gender. Results indicated that moral distress was negatively correlated with moral resilience (*β* = −0.184, *t* = −4.378, *p* < 0.01), while moral courage was positively correlated with moral resilience (*β* = 0.041, *t* = 5.821, *p* < 0.01). Moreover, in the moderated mediation model, the interaction term moral distress × moral courage was significant (*β* = 0.001, *t* = 3.343, *p* < 0.001), indicating that moral courage moderated the relationship between moral distress and moral resilience, as shown in Table 5 and Figure 2. Thus, Hypothesis 2 was supported. To analyze the differential effects of moral courage, this study conducted a simple slope test categorizing moral courage into high levels (one standard deviation above the mean) and low levels (one standard deviation below the mean). When moral courage levels were low, moral distress was significantly negatively correlated with moral resilience (*β* = −0.773, *t* = −5.873, *p* < 0.001). When moral courage levels were high, the negative correlation of moral distress with moral resilience was not significant (*β* = −0.189, *t* = −1.469, *p* > 0.05). Simple slope analysis confirmed that moral courage buffers the link between moral distress and moral resilience: the relationship was significant at low levels of moral courage but not at high levels, as shown in Figure 3.

**Table 5:** Results of the moderated mediation model.

Variables	moral resilience			burnout		
	*β*	SE	*t*	*β*	SE	*t*
Constant	46.954	2.24	20.958	–	–	–
Moral distress	−0.184	0.042	−4.378^**^	0.075	0.027	2.771^**^
Moral courage	0.041	0.030	5.821^**^	–	–	–
Moral resilience	–	–	–	−0.556	0.142	−3.916^**^
Moral distress × moral courage	0.001	0.006	3.343^***^	–	–	–
*R* ^2^	0.156			0.083		
*F*	20.302^***^			14.939^***^		

^**^p < 0.01, ^***^p < 0.001.

## 4 Discussion

This study explored the relationship between moral distress and burnout among emergency nurses in China, building upon existing theoretical frameworks and previous research. It emphasizes the mediating role of moral resilience and the moderating role of moral courage. The findings revealed a significant positive correlation between moral distress and burnout. Furthermore, moral resilience was identified as a partial mediator in this relationship, while moral courage moderated the link between moral distress and moral resilience. These results contribute to a deeper understanding of the relationship between moral distress and burnout among emergency nurses, providing empirical evidence to support strategies aimed at enhancing psychological well-being, work engagement, and the quality of care in emergency nursing settings.

### 4.1 Relationship between moral distress and burnout

Our study revealed a positive correlation between moral distress and burnout, consistent with previous findings (24). The emergency department is characterized by a fast-paced environment and high levels of uncertainty, with nurses constantly exposed to traumatic and distressing events such as severe injuries, death, and suicide (25). Emergency nurses often face verbal and physical aggression from patients and their families (26). As a result, nurses in the emergency department are more likely to experience burnout than nurses in other departments (27). Emergency nurses often face uneven distribution of resources and a lack of understanding from patients and their families regarding treatment decisions. Most emergency nurses have experienced physical or non-physical violence, and the convergence of these multiple factors leaves them facing greater moral distress (2, 3). A recent cross-sectional study has confirmed that moral distress was significantly associated with burnout and turnover intention among nurses (7). Therefore, deeply understanding and effectively addressing moral distress have become crucial to identifying factors linked to burnout and sustaining the quality of care.

### 4.2 The mediating role of moral resilience

This study demonstrated that moral resilience partially mediated the relationship between nurses’ moral distress and burnout, supporting Hypothesis 1 and consistent with prior research (7). It is particularly noteworthy that this study found the mediating effect of moral resilience accounted for as much as 24.8% of the total effect. In the first stage of the mediation pathway, moral distress was significantly negatively associated with moral resilience (*r* = −0.238, *p* < 0.01), indicating that higher moral distress is associated with lower moral resilience. This is consistent with previous research (28). In the second stage, moral resilience was also negatively associated with burnout (*r* = −0.248, *p* < 0.01), further supporting the notion that fostering moral resilience is essential for effectively mitigating burnout, a finding consistent with previous studies (11). Specifically, greater exposure to moral distress is associated with lower moral resilience, which, in turn, is related to higher burnout.

Previous studies (7) applied a similar framework but used instruments validated for general healthcare workers, limiting reliability in certain dimensions. By contrast, this study employed a tool specifically designed for registered nurses, ensuring a targeted sample and items aligned with nursing practice, as supported by a recent Chinese review (10). These findings provide robust evidence that moral resilience mediates the relationship of moral distress on burnout among emergency nurses.

### 4.3 The moderating role of moral courage

This study examined the moderating role of moral courage in the relationship between moral distress and moral resilience among emergency nurses, supporting Hypothesis 2. We found that moral courage plays a moderating role in the first half of the mediation process, suggesting that moral courage moderated the relationship between moral distress and moral resilience. Moral courage was identified as a protective factor for moral resilience, consistent with previous research (5). According to the theory of moral resilience, moral resilience, defined as the ability to maintain or restore moral integrity in the face of ethical challenges, is fundamentally supported by moral courage (29). Nurses with higher moral courage are more adept at identifying core conflicts in moral distress, enabling them to mobilize resources and sustain moral resilience (30). Given these findings, nursing administrators and educators should consider interventions to enhance moral courage among emergency nurses, thereby supporting moral resilience and promoting psychological well-being.

While previous studies have explored moral resilience and moral courage as serial mediators, this study contributes by examining moral courage as a moderator in the relationship between moral distress and moral resilience.

### 4.4 Research strengths

This study has several notable strengths. First, the Moral Courage Scale and the Rushton Moral Resilience Scale assess different ethical-related capacities, supporting their appropriateness for concurrent application in this model. Second, we adopted the Chinese version of the Rushton Moral Resilience Scale for its favorable psychometric properties and better clinical comprehensibility among participants. Third, moderated mediation analysis was used as it is well-suited for testing the proposed hypothetical pathways. Fourth, by incorporating moral courage as a moderator, this study extends previous findings and enhances the novelty of the research model.

## 5 Limitations

This present study has several limitations. First, the cross-sectional design precludes causal conclusions, as the findings reflect associations rather than causal effects. Longitudinal or experimental studies are needed to establish temporal relationships and rule out alternative explanations. Thus, the observed mediation effect should be viewed as an indirect relationship rather than a causal mechanism. Second, the sample consisted of emergency nurses from the Ningxia Hui Autonomous Region, which limits the generalizability of the findings. Multicenter studies are necessary to confirm these results across diverse populations. Finally, the study was conducted in a single cultural context (China), and the generalizability of the findings to other healthcare systems and cultures requires further investigation.

## 6 Conclusion

In summary, our study examined the association between moral distress and burnout among emergency nurses, testing the mediating role of moral resilience and the moderating role of moral courage. Findings indicated a positive association between moral distress and burnout, with moral resilience partially mediating this link and moral courage moderating the path from moral distress to moral resilience.

## Data Availability

The original contributions presented in the study are included in the article/supplementary material, further inquiries can be directed to the corresponding author.
